# The Treatment of Retention of Urine, Etc.

**Published:** 1877-12

**Authors:** Jno. Thad. Johnson

**Affiliations:** Atlanta, Ga., Professor of Anatomy and Lecturer on Venereal Diseases in the Atlanta Medical College


### THE TREATMENT OF RETENTION OF URINE, WHEN CAUSED BY STRICTURE OF THE URETHRA.

By JNO. THAD. JOHNSON, M.D., Atlanta, Ga.,
Professor of Anatomy and Lecturer on Venereal Diseases in the Atlanta Medical College.

(*Essay read before the Atlanta Academy of Medicine.*)

To treat retention of urine intelligently, it is essential that the nature of the obstacle be discovered. I shall speak here of that retention produced by stricture, because of its greater frequency, and especially because of the difficulty in relieving it, unless the proper instruments, and the requisite tact in their manipulation, are at the command of the physician. I do not say that any vast array of instruments is necessary. Most surgical operations may, under emergency, be performed with temporized devices. We rather admire the readiness and adaptability of the operator who, if necessary, finds a hand-saw, a pocket-knife, and pair of tweezers, sufficient for all amputations. But the urethra being a canal dark, deep, and narrow, some special appliances for relief of its obstructions are indispensable. An ordinary gum or silver catheter, the time-honored doctor's companion, will reach the bladder through a normal urethra; but, for passing through a tight stricture, it is about as efficient as a crow-bar. Very much to be pitied is that doctor who, armed with such a catheter, and far removed from better appliances or professional assistance, is called upon to remove a bladder-full of urine accumulated behind a tight stricture. He probably feels it incumbent on him to persist in his efforts at introduction; and these efforts may but serve to prolong the spasm or congestion that has occasioned the distress. I am not sure whether doctor or patient suffers the greater distress.

These attacks of retention occur with patients who have suffered for a greater or less time with a tight organic stricture, through which the urine could be forced only in a small stream. From some of many familiar exciting causes, the condition of spasm, or of congestion, or both, has been superadded, and what of the canal was heretofore pervious, is, for a time, obliterated. It is important that this mode of causation be recognized, as a knowledge of it will be serviceable in our treatment.

Most naturally, the first attempt for the relief of a patient suffering from retention, will be to withdraw the fluid *per urethram.* But it should be borne in mind that these attempts should be always of the gentlest character. Not only should the instrument be carefully handled, but it should be of the material and construction least liable to penetrate the mucous membrane of the urethra. Metal instruments of small calibre are dangerous, even in skillful hands. They are not only dangerous, but generally useless, for they do not offer the best hope of threading a narrow canal.

The small olive-pointed catheter or bougie, of French make, (though the English answers very well) with a slender and flexible neck, is comparatively safe and generally efficient. But unless the neck of the instrument be slender and flexible, it is but little better than the old-fashioned gum catheter.

Should the smallest size of the olive-pointed instrument prove unavailing, resort should be had to the filiform whalebone bougie. By gentle manipulation and rotation, one of these, very likely, will reach the bladder, and allowing it to remain for some little time, its withdrawal will probably be followed by the urine. Or, having introduced one, a second or even a third may be passed by the side of it. This may be accomplished more readily by pushing the first on until the *swell* or largest part is in the stricture, then withdrawing it until its neck or smallest part corresponds with the obstruction, and then insinuating the second by its side.

Now, while every physician is not expected to be provided with a formidable armamentarium, it will not be found expensive to have on hand such appliances as are enumerated above; or, if he is more liberally provided, a tunneled catheter may be introduced over the filiform. It is not always easy, though, to slide a tunneled instrument through a narrow canal over a bougie, (*guide*) for the catheter must necessarily be of considerable size.

A better plan, as I think, for following a filiform with a catheter, consists in using the gum filiform, tipped with a screw, for connecting to a corresponding screw in the end of a small silver catheter, on the principle of the staff of Maisonneuve's urethrotome. Some time since I had Mr. W. F. Ford, of New York, to construct such a catheter for me. Since then, I learned that such an instrument was already in use.

It is scarcely necessary to say that our patient should be placed in the best condition for relaxing what of spasm of the urethra may be present. The prolonged warm bath, the hot blanket, a sweat bath, opium freely given, and even anæsthesia, should be resorted to. Certainly diuretics should not be used, as I saw recommended some time since, for relieving retention. The bladder has already more water than it has use for. When these measures, I say, have been tried—when the instruments at hand have been judiciously used, but without avail, then resort should be had to other means of relief. To continue the punching at the stricture, and probably with an ill-adapted instrument, can do only harm. It would tend to keep up, even to increase, that spasm and congestion to which the sudden stoppage of urine is attributable. And if it is not possible to aspirate, or make other operation, it is much better practice to quiet your patient with opium *per orem* or hypodermically. This will relax the spasm and prevent, to a large degree, the further distension of the organ, by preventing the elimination of much water from the kidneys. Opium, in full doses, is better than *injudicious* attempts at catheterism. If, however, the retention has already lasted so long that the patient exhibits a tendency to coma, we should be extremely careful in the use of this remedy.

But it is not proper to allow the bladder to remain so greatly distended for many hours, with such possible dangerous consequences, and with so much suffering for the patient. We should resort to puncture. The trocar through the rectum or above the pubes, until a recent date, were the means of puncture at our command. The aspirator now serves its purpose more kindly, and being easily managed, and almost free from danger, there is less hesitation in resorting to it.

An operation which I believe better than puncture with a trocar over the pubes, or through the rectum, and one which might be executed in the absence of an aspirator, consists in plunging a bistoury through the perineum into the distended urethra, behind the stricture, and just in front of the prostate gland. This can be done, when the bladder is distended, without great difficulty, by the aid of the finger in the rectum. The propriety of extending the incision forward from this point to divide the stricture, constituting external perineal urethrotomy, will be presently discussed.

It is always proper for the surgeon, when called upon to relieve retention incident to a stricture, to consider if it is not better to make some operation which will cure the stricture as well as relieve the present distress of the bladder. Otherwise, in all likelihood, when the patient has been relieved, he will postpone measures for a cure, and thus remain in constant danger of a recurrence of his trouble, and of other evils whose approach is not less dangerous because more insidious. But we should consider, further, whether an operation would be more dangerous because of the condition of the patient, than it would be by waiting for some days or weeks for recovery from the prostration incident to retention.

If we have succeeded in introducing a filiform bougie, we might be tempted to follow it with a divulsor, and so open the stricture. This, I believe, would not be good practice. The shock from divulsion is greater than from any other method of operating. This is true of the measure, even when undertaken with a patient in good condition, and the danger is intensified from the suffering and depression in the case we now consider.

Then we are left to choose between internal and external urethrotomy. If we have succeeded in passing the guide to the bladder, we can follow it, very likely, with Maissonneuve's urethrotome, and so divide the stricture by internal urethrotthrotomy. To make the operation still more thorough—that is, to make *complete division* of the stricture bands—Otis' instrument should supplement the one already used.

For the above reasons, then, if we have decided to operate at once for the stricture, we may proceed as above directed, provided any operation from *within the urethra* be admissible. But I think it may be laid down as a rule, that patients suffering from prolonged retention do not bear operations within the canal so kindly as external urethrotomy. The more prostrated the patient, or the greater the disease of the bladder, so much the more, I believe, is "perineal section" to be preferred to division from within the urethra. It is not my object here, nor is it within the scope of this paper, to treat of the ease with which "perineal section with a guide" may be made, nor of the best methods of effecting "perineal section without a guide;" but I would urge the value of this operation when we have decided to relieve at a stroke both retention and its cause.

As above stated, internal urethrotomy is to be preferred to divulsion; but external urethrotomy (if the stricture be in the deep urethra) is to be preferred to the internal incision in those cases greatly prostrated by either the retention or other consequences of stricture.

